# Morphological Evidence Supports the Taxonomic Reinstatement of the Endemic Chinese Species *Iris pandurata* (Iridaceae) by Segregation from *I. tigridia*

**DOI:** 10.3390/plants13233418

**Published:** 2024-12-05

**Authors:** Eugeny V. Boltenkov

**Affiliations:** Botanical Garden-Institute, Far Eastern Branch, Russian Academy of Sciences, 690024 Vladivostok, Russia; boltenkov@rambler.ru

**Keywords:** China, *Iris trippneriana*, morphology, narrow endemic species, nomenclature, Qilian Mountains, synonym, taxonomy

## Abstract

Unfortunately, the statuses of the numerous names of the genus *Iris* at specific rank remain unresolved. This study considers *I. pandurata*, the species that was first described in 1880 and then 30 years later and is, however, still synonymized with *I. tigridia*. The morphological relationship between these two species and *I. trippneriana*, a previously unplaced name, has been assessed here. The morphological analysis has revealed that *I. pandurata* and *I. trippneriana* are actually a single species that can easily be distinguished from *I. tigridia* by the rhizome shape, the adventitious roots shape, the flowering stem structure, and the falls ornamentation. The results support the reinstatement and recognition of *I. pandurata* at specific rank, including *I. trippneriana* as a synonym. Being endemic to China, *I. pandurata* has a narrow distribution range in the Qilian Mountains in the Gansu and Qinghai provinces. This report provides an updated nomenclature for *I. pandurata*, including *I. tigridia*, detailed photographs of living plants to facilitate identification, images of type specimens, a list of specimens examined, and comments on their distributions and habitats. A lectotype for *I. trippneriana* is designated here.

## 1. Introduction

**Figure 1:**
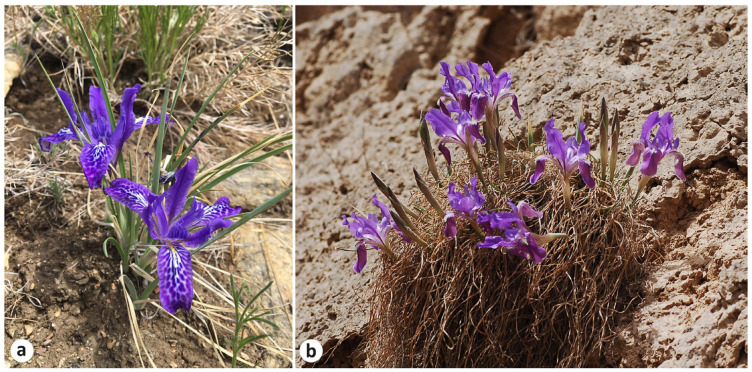
Habit of *Iris* species studied: (**a**) *I. tigridia* (Russia, Republic of Buryatia, Kyakhtinsky District, 10 km east of Kyakhta), photographed by the author; (**b**) *I. pandurata* (China, Gansu Province, Yuzhong County), photographed by J. Pan.

The abundance of unreliable information published in the literature and websites as regards the genus *Iris* L. (Iridaceae) continues to confuse its actual taxonomic diversity and highlights how critical it may be [1,2,3]. Recently [4], it has been confirmed that *I. ivanovae* Doronkin, previously reported for the Republic of Buryatia and Zabaykalsky Krai, Russia, is a synonym of *I. tigridia* Bunge. However, it is not the only synonym of *I. tigridia* according to the literature and dedicated websites. Numerous sources indicate that *I. pandurata* Maxim. is also a taxonomic synonym of *I. tigridia* [5,6,7,8,9,10,11,12,13,14,15,16,17,18,19,20,21,22,23]. On the other hand, *I. pandurata* has been accepted in references [24,25,26,27,28,29,30,31,32,33,34,35,36,37]. In China, the common names of *I. tigridia* are ”Thick-rooted iris” (or 粗根鸢尾 “Cu gen yuanwei” in Chinese) and ”Pseudo-tiger iris” (or 拟虎鸢尾 “Ni hu yuanwei” in Chinese), while *I. pandurata* is referred to as “Gansu iris” (or 甘肃鸢尾 “Gansu yuanwei” in Chinese) [7,8,14,29] (Figure 1).

*Iris pandurata* was first described based on plants collected by Nikolai Mikhailovich Przhevalsky in his first journey to Central Asia (1870–1873) on the way from Qinghai Lake to the mountains south of the Datong River in late May 1873 [38] (p. 235). This was the time when Przhevalsky explored the eastern part of the Qilian Mountains northeast of the present city of Xining (Qinghai Province, China). Farrer [39,40] called this locality the Da-Tung Alps. *Iris tibetica* (Dykes) Bolt., an endemic to China, was described from there as well [41].

Carl Johann Maximowicz, the author of the name *I. pandurata*, distinguished this species from *I. tigridia* only by the two-flowered stem in the Latin diagnosis [42]. Dykes [6] considered this feature characteristic of both *I. tigridia* and *I. pandurata* and synonymized the latter with *I. tigridia*. However, based on my examinations of the protologues [42,43], original material, the relevant literature [25,31,32,35], and recent molecular data (sub *I. tigridia*) [44,45], I hypothesized that *I. pandurata* is a distinct species.

In this study, I used extensive morphological data to re-evaluate the relationship between *I. tigridia* and *I. pandurata*. The aim was to determine whether *I. tigridia* and *I. pandurata* represent two distinct species or both names refer to a single species. When preparing this article, I paid attention to *I. trippneriana* E.Peter [46] that had been described from the same area as *I. pandurata*. According to the databases [15,17,20], this name is unplaced because the type material has not been studied and is awaiting taxonomic scrutiny. Therefore, it cannot be accepted, nor can it be put into synonymy. Thus, an additional aim was to study the taxonomy of *I. trippneriana* and establish the species to which the concept of the name belongs.

## 2. Results

**Figure 2:**
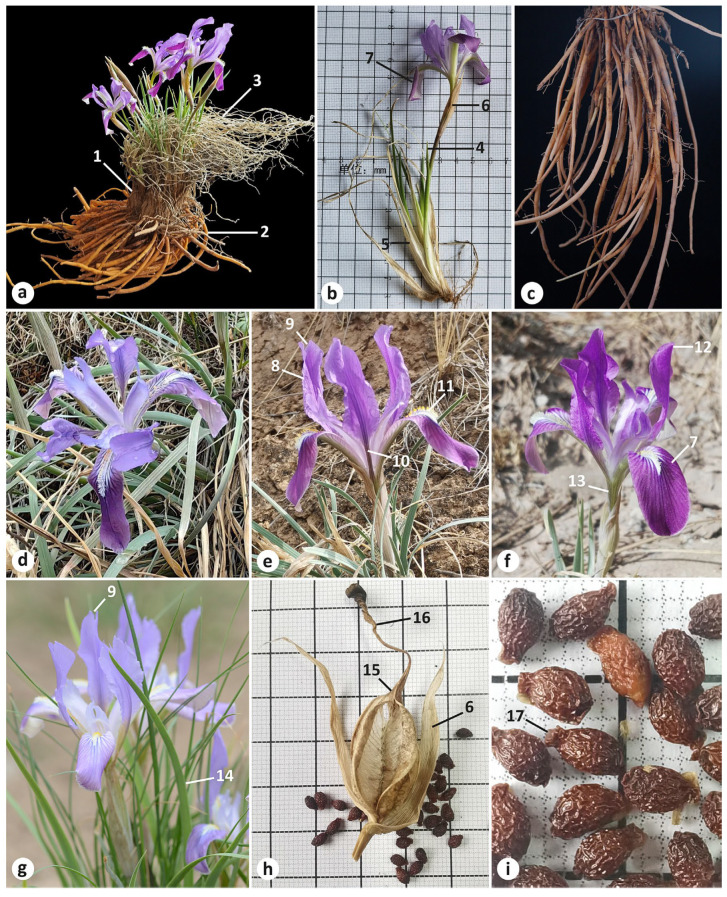
Morphological characters of *Iris pandurata* from China: (**a**) habit; (**b**) flowering stem ((**a**,**b**) Qinghai Province, Xining City, Chengdong District); (**c**) adventitious roots (Gansu Province, Yuzhong County); (**d**–**g**) flowers ((**d**) Gansu Province; (**e**) Qinghai Province, Xining City, Danan Mountain; (**f**) Qinghai Province, Xining City, Chengdong District; (**g**) Gansu Province, Lanzhou City, Anning District); (**h**) fruit and seeds; (**i**) seeds ((**h**,**i**) Qinghai Province, Xining City, Nanyou Mountain). (**a**,**b**,**e**,**f**,**h**,**i**) photographs were taken by S. Zhao; (**c**) by J. Pan; (**d**) by C. Song; and (**g**) by L. Liu. Numbering is as follows: 1, leaf remnants; 2, adventitious root; 3, dry rosette leaves; 4, cauline leaf; 5, basal leaf; 6, outer bract; 7, blade of fall rounded at the apex; 8, standard emarginate at apex; 9, notch; 10, claw; 11, beard; 12, standard rounded at apex; 13, perianth tube; 14, rosette leaf; 15, beak; 16, perianth persistent; 17, aril.

**Figure 3:**
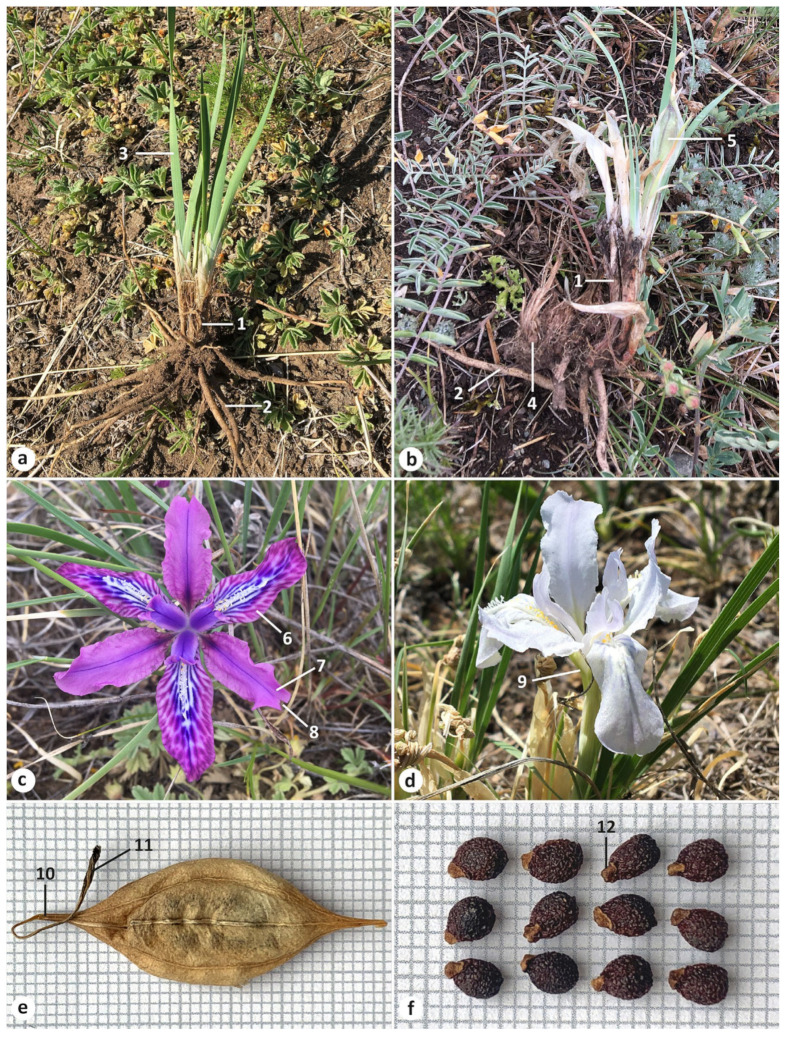
Morphological characters of *Iris tigridia* from Russia: (**a**,**b**) habit; (**b**) habit ((**a**) Zabaykalsky Krai, Mogoytuysky Raion, vicinity of Aga Village; (**b**) Altai Republic, Ust-Kansky Raion, vicinity of Tiudrala Village); (**c**,**d**) flowers ((**c**) Republic of Buryatia, Kyakhtinsky Raion, 10 km east of Kyakhta; (**d**) Zabaykalsky Krai, vicinity of Khara-Byrka Village); (**e**) fruit (Republic of Buryatia, Kudara River basin); (**f**) seeds (Republic of Buryatia, Selenginsky Raion, confluence of Selenga and Chikoy). Photographs were taken by the author. Numbering is as follows: 1, leaf remnants; 2, adventitious root; 3, rosette leaf; 4, rhizome; 5, fruit; 6, blade of fall; 7, standard emarginate at apex; 8, notch; 9, perianth tube; 10, beak; 11, perianth persistent; 12, aril.

**Figure 4:**
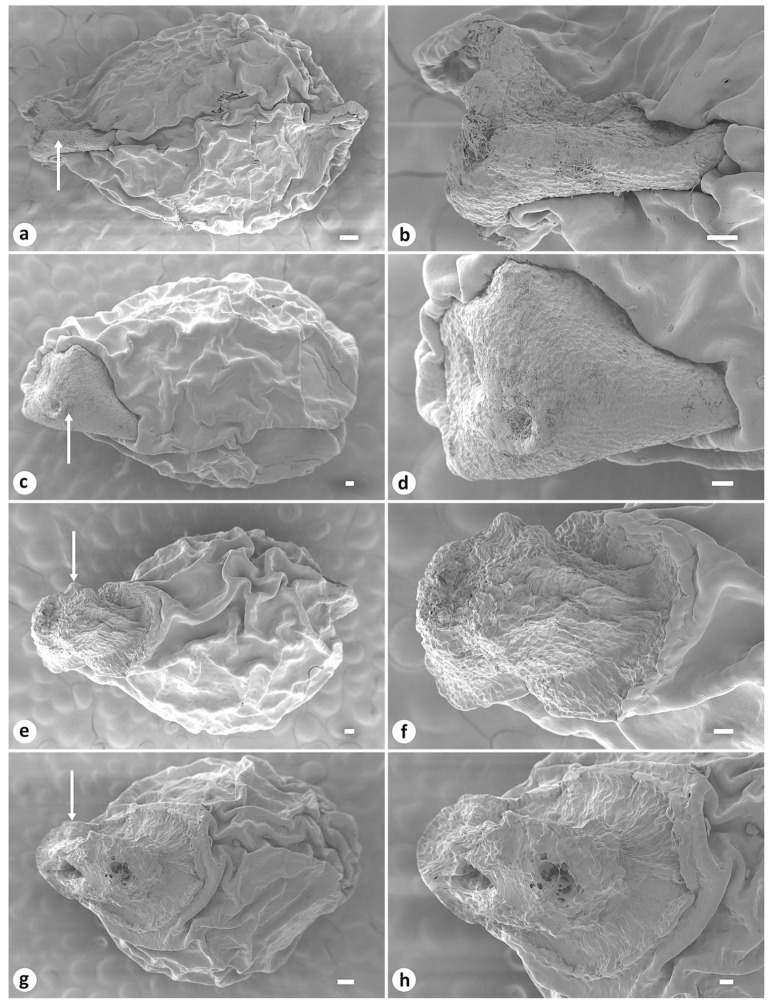
Scanning electron micrographs of seeds of the *Iris* species studied: (**a**–**d**) *I. pandurata* ((**a**,**b**) China, Gansu Province, 67 km north of Lanzhou; (**c**,**d**) Gansu Province, Lanzhou, Yuquan Mountain); (**e**–**h**) *I. tigridia* ((**e**,**f**) Russia, Republic of Buryatia, Kudara River basin; (**g**,**h**) Russia, Tuva Republic, vicinity of Bayan-Kol). White arrows indicate aril. Scale bars: (**a**,**g**) 200 μm; (**b**–**f**,**h**) 100 μm.

A morphological comparison between *I. pandurata*, *I. tigridia*, and *I. trippneriana* is listed in Table 1 (see also Table S1 for more details). The morphological features of *I. pandurata* and *I. tigridia* are presented in Figure 1, Figure 2, Figure 3 and Figure 4. These species shared the features listed below. All are perennial herbs, with straight fibrous remnants of leaf bases around the stems. Their adventitious roots were yellowish brown, vermiform, thickened, and up to 0.4 cm in diameter, with few small lateral roots. Rosette leaves were ensiform and rarely subfalcate with a narrowly acute and straight apex, tough, and finely ribbed. The flowering stem was shorter than the rosette leaves and bore a terminal cluster (simple), a cauline leaf, membranous basal leaves which were wider than the rosette leaves, two membranous, lanceolate bracts with a narrowly acute apex and distinct parallel brown veins, and a subsessile pedicel. The flowers were similar in diameter and variable in color. The outer perianth segments (falls) were gradually narrowed into a claw, and the inner perianth segments (standards) were abruptly narrowed into a thin claw. The blades of the falls were obovate with a beard of white, yellow-tipped hairs. The standards were oblong, lacked hairs, and were with or without a distinct sharp notch at the apex (emarginate), monochromatic with dark-colored veins.

In addition, in *I. pandurata* and *I. tigridia*, the rosette leaves were glaucous. Fruit was a fusiform capsule with dehiscence loculicidal below the apex. The plants were always with dry perianth persistent or with a beak 0.2–0.5 cm long. The seeds were pyriform, reddish brown, ca. 4.6 mm long and 3 mm in diameter, and bore yellowish white arils. The seed surface was glossy and wrinkled (rugose), with testa that was more or less hard.

*Iris pandurata* and *I. trippneriana* were morphologically quite clearly distinguished from *I. tigridia* by the compact, vertical rhizome (see https://www.cvh.ac.cn/spms/detail.php?id=de40d6a8, accessed on 13 October 2024) and was up to 1.5 cm long (vs. shortly creeping, horizontal rhizome; see Figure 3b). They had numerous adventitious roots, which were crowded, with the upper part being even in thickness and gradually tapering to the apex, smooth, without transverse patterns, but longitudinally wrinkled in dry, very long, up to 27 cm long (vs. adventitious roots thickened in the upper part, with wrinkled transverse patterns, or so-called “contractile”, elongated, up to 15 cm long). The rosette leaves were slightly narrower, up to 0.4 cm long (vs. up to 0.6 cm long). The flowering stem, which bore mainly two flowers, also featured a well-developed secondary bract (bracteole), with the bracts and bracteole were equal in length (vs. a single flower, without a bracteole). The perianth tube was surrounded by bracts that often extended to the middle of the claw of falls, unlike those of *I. tigridia*, which are typically not longer than the perianth tube. The blade of the falls was folding downwards and unspotted with a pattern of purple veins, and the apex was not emarginate (vs. spreading almost horizontally, spotted, and with darker lines, with the apex being occasionally emarginate). In addition to these differences between *I. pandurata* and *I. tigridia*, arils of the former were small and beak-shaped (vs. mushroom-shaped arils; see Figure 4). In the wild, plants of *I. pandurata* are surrounded by a lot of prostrate, wavy, dry rosette leaves (Figure 2a).

## 3. Discussion

### 3.1. Taxonomy of Iris pandurata

The morphological analysis has shown that *I. trippneriana* belongs to *I. pandurata*, a species certainly distinct from *I. tigridia*. *Iris pandurata* may easily be distinguished from *I. tigridia* by the compact rhizome; by the evenly thickened, smooth, very long and numerous adventitious roots; by the two-flowered inflorescence with three bracts; and by the unspotted falls. Earlier, it was reported that seeds of *I. pandurata* lacked appendages [16,28,29,36]. In the present study, I have found that seeds of *I. pandurata* are arillate. Seeds of *I. pandurata* can be distinguished from *I. tigridia* by their small, beak-shaped aril (Figure 4a–d). Plants with such characteristics are distributed in the eastern Qilian Mountains between eastern Qinghai Province and central Gansu Province, China. Thus, the present taxonomic investigation will contribute to our knowledge of the Chinese species, particularly the identity of *I. pandurata*, a narrow endemic species to China.

I have managed to determine the source of the taxonomic confusion related to *I. pandurata*: it arose from reference [5], where Dykes noted, with respect to the species, that “specimens from the natural habitat show that both one and two-flowered stems are borne by the same individual plants, and it seems probable that it is really only a strong-growing local variety of *I. tigridia*”. In his monograph [6], Dykes synonymized *I. pandurata* with *I. tigridia* by stating that “there seems to be no ground for separating” these species. Farrer [39,40] and Dykes [47], following their statements, attributed Farrer’s gathering No. 498 from the Da-Tung Alps to *I. tigridia* (see Supplementary Data in File S1).

However, some botanists did not support Dykes’s view. For instance, Grubov [25] claimed that *I. pandurata* was a “good” species with stable characters. Yu-Tang Zhao, a prominent expert in the Chinese *Iris* species, initially also accepted *I. pandurata* [26,27,28,29]. He rightly noted that *I. pandurata*, which had been mistakenly regarded as a synonym of *I. tigridia* by Dykes [6], can easily be distinguished from the latter by its two-flowered stem; by its longer adventitious roots, which are almost equally thickened at the proximal and distal parts; and by the distribution in the Gansu and Qinghai provinces vs. the single-flowered stem; relatively thick adventitious roots bases, which gradually taper to the apex, with wrinkled transverse patterns; and the distribution in Northeast China and Inner Mongolia [28,29,32]. Rix [35] also noted that *I. pandurata* can be distinguished from *I. tigridia* by its adventitious roots with a uniform diameter down their length. However, unfortunately, *I. pandurata* was synonymized with *I. tigridia* in the *Flora of China* [7], which is the authoritative source for Chinese botanists.

On the basis of such features as a compact rhizome, unbearded standards, and a slightly noticeable aril, *I. pandurata* is attributed here to *I*. sect. *Pseudoregelia* Dykes, which is consistent with references [30,32,35]. In the present contribution, I have found genetic support for this sectional affiliation. The evidence is the OR774963 accession from the complete chloroplast genome sequence (sub *I. tigridia*) [13] of a specimen originally collected in Gansu Province, China (S. Volis, pers. comm.). This accession forms a distinct monophyletic clade with *I. goniocarpa* Baker and *I. sichuanensis* Y.T.Zhao, the species of *I*. sect. *Pseudoregelia* Dykes. Although the OR774963 accession is named *I. tigridia* in references [44,45], it can reasonably be named *I. pandurata* based on its morphology (Figure 2d) and distribution range. Nevertheless, our phylogenetic analyses unambiguously indicated that *I. tigridia* belongs to unispecific *I*. ser. *Tigridiae* Doronkin of *I*. sect. *Psammiris* (Spach) J.J.Taylor rather than to *I.* sect. *Pseudoregelia* [4,48].

### 3.2. Taxonomic Treatment

**Figure 5:**
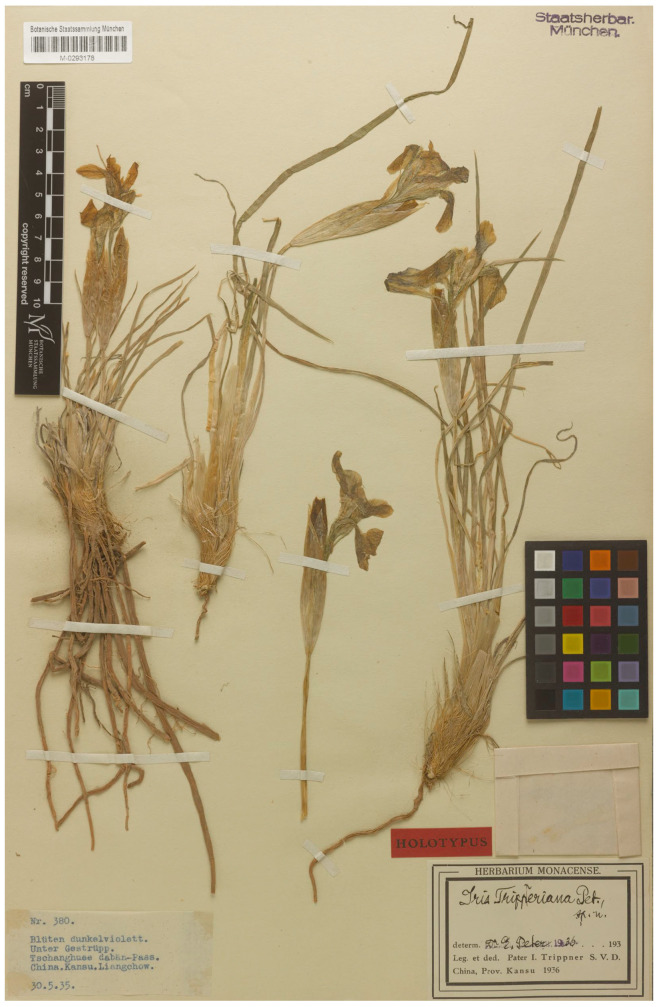
Lectotype of *Iris trippneriana* (M0293178) (included with the permission of the curator).

There are some comments concerning the type citation of the names under study. Below are a number of details that should be clarified:

(i) In the protologue of *I. pandurata*, the locality (“prov. Kansu”) and the collector’s name (“Przewalski”) were mentioned as follows: “China occidentalis, prov. Kansu, ad rupes praeruptas rarissima (Przewalski)” [42]. No specimen or gathering was indicated in the protologue because there was no direct reference to the collecting date or number (Art. 40, Note 2 of the *Shenzhen Code* (hereafter ICN, [49]). Grubov [25] (p. 99) cited the specimen from LE, collected from Changtang, a high-altitude plateau in western and northern Tibet including the southeastern regions of Ladakh, as follows: “Yuzhno-Tetungskiy khrebet, nizhniy poyas, na krutykh skalakh, ochen’ redko [South Datung ridge, the lower belt, on steep rocks, very rare], 29 May 1873, Przhevalsky, typus! [originally in Russian]”. This specimen belongs to the collection of Przhevalsky from his first journey to Central Asia. It is likely that only a single specimen has ever existed (which, in this case, would be the holotype), which, however, cannot be established for certain because the name *I. pandurata* was published without a holotype. Thus, in citing “typus”, Grubov designated the specimen in LE (LE01011518) as the lectotype of *I. pandurata*, thus satisfying the requirement of the ICN (see Art. 9.1, Note 1).

(ii) *Iris trippneriana* was described by Elfriede Peter [46], an Austrian botanist. The plants were collected in the 1930s from the Tschanghue-daban Pass in the vicinity of Lanzhou, Gansu Province, Northwestern China, by Joseph Trippner, a Catholic missionary of the Steyler Mission in Lanzhou who served in the Kokonor area, often referred to as northeastern Amdo, present China’s Qinghai Province [50]. In the protologue of *I. trippneriana*, a single gathering in two herbaria (M and W) was cited as follows: “W-Kansu: Tschanghue-dabän Paß bei Liangtschou, unter Gestrüpp, 30.V.1935 (Trippner 380: Herb. München, Herb. Mus. Wien)” [46]. Therefore, there must exist at least two duplicate specimens, which are syntypes (Art. 40, Note 1 of the ICN). Unfortunately, the specimen at W has not been found to date (J. Walter and H. Rainer, pers. comm.). It is probably a part of the monocots collection of the Natural History Museum (Vienna, Austria) that was lost during World War II. As a consequence, the preserved specimen, i.e., M0293178 (Figure 5), is designated here as the lectotype.

According to the circumscription presented, *I. tigridia* and *I. pandurata* are accepted here, with the latter reinstated as a distinct species. Information on these two species (highlighted in bold italics) is provided below with their synonyms, full nomenclature citations, illustrations, and the main findings on their distributions and habitats.

***Iris tigridia*** Bunge, Fl. Altaic. [Ledebour] 1: 60, 1829.—Lectotype (designated by Boltenkov [4] (p. 26)): [Russia, Altai Republic] Altai, in schistosis ad fluvium Tscharysch, [4 May] 1826, *Bunge 50*, Herb. C.A. Meyer (LE01010797!).—http://re.herbariumle.ru/01010797 (accessed on 13 October 2024).

= *Iris ivanovae* Doronkin, Fl. Sibir. (Arac.-Orchidac.) 4: 117, 1987.—Holotype: [Russia, Zabaykalsky Krai] Chita Oblast, Borzinskiy District, Kharanor, feather-grass steppe, 7 June 1965, *A. Zarubin s.n*. [originally in Russian] (NSK0000077!).—https://www.jacq.org/detail.php?ID=525145 (accessed on 13 October 2024).

Illustrations of *I. tigridia*: [28] (t. 62, f. 3–4), [29] (t. T, f. 3–4), [51] (t. 342), [52] (t. 107, f. 3–4), [53] (t. 389, f. 1).

Distribution and habitat: In China, *I. tigridia* is distributed in Beijing, Inner Mongolia Autonomous Region, and the Hebei, Jilin, Liaoning, and Shanxi provinces [4] (also see File S1). It is also found in southern Siberia, Russia, eastern Kazakhstan, and northern and central Mongolia. It grows in gravelly, stony, or sandy habitats in steppes among grasses and also on dunes, rocky slopes, and hilltops. The flowering season is from April to early June; the fruiting season is from May to July.

Previously, we reported that *I. tigridia* was found at elevations ranging from 400 to 1200 m [4]. However, it actually has a wider elevation distribution range: from 136 to 2500 m (see File S1). I found *I. tigridia* in the Khangai Mountains (vicinity of Khujirt, central Mongolia) at an elevation of 1810 m. The Chinese herbarium specimens of *I. tigridia* from Shanxi Province were collected at elevations of 2200 and 2500 m (PE01013763 and PE01013768, respectively; see File S1).

**Figure 6:**
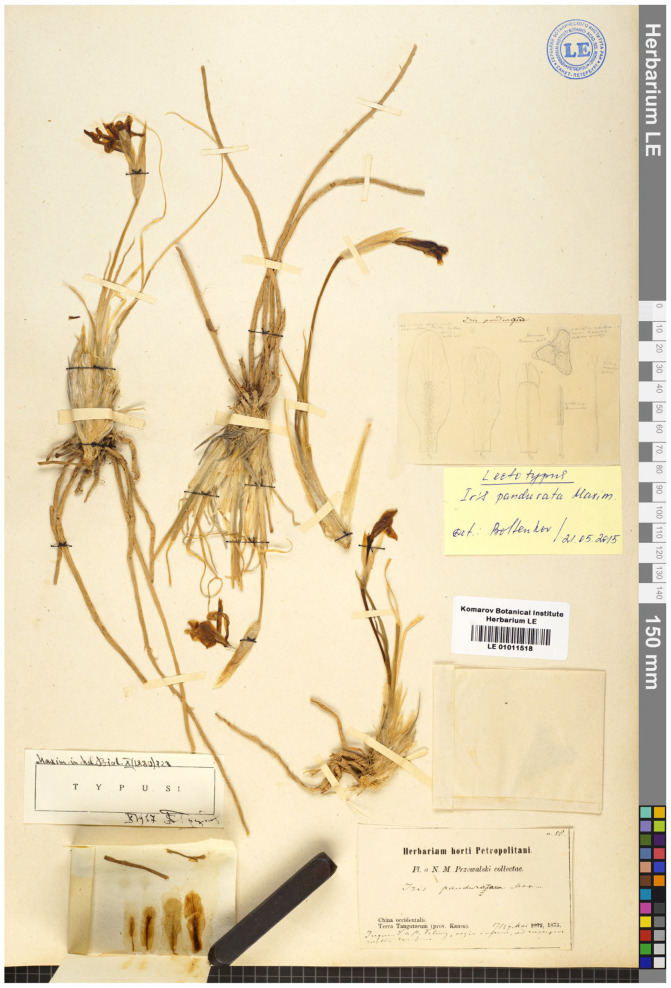
Lectotype of *Iris pandurata* (LE01011518) (included with the permission of the curator).

***Iris pandurata*** Maxim., Bull. Acad. Imp. Sci. Saint-Pétersbourg 26(3): 529, 1880.—Lectotype (designated by Grubov as “typus” [25] (p. 99)): [Qinghai Province] China occidentalis, Terra Tangutorum (prov. Kansu), Jugum S. a fl. Tetung [Datong River], region inferior, ad rupes praeruptas rarissima, 17/29 May 1873, *N.M. Przewalski 58* (LE01011518!, cum icon. auct.). Figure 6.

= *Iris trippneriana* E.Peter, Oesterr. Bot. Z. 87(2): 129, 1938, ***syn. nov.***—Lectotype (designated here): [Label 1] China, Kansu, Liangchow, Tschanghuee dabän-Pass, unter Gestrüpp, Blüten dunkelviolett, 30 May [19]35, Nr. *380*; [Label 2 with the printed note “Herbarium Monacense”] *Iris trippneriana* Pet., *sp. n*. Determ. Dr. E. Peter, 1936, Leg. et ded. Pater I. *Trippner* S.V.D. [i.e., Societas Verbi Divini, the German-Dutch Catholic Congregation] China, Prov. Kansu 1936 (M0293178 [digital image!]). Figure 5.

Illustrations of *I. pandurata*: [25] (t. 6, f. 5), [28] (t. 60, f. 5–6), [29] (t. R, f. 5–6), [33] (f. 485), [54] (t. 87, f. 7–8), (sub *I. tigridia*) [55] (t. 356, f. 5–6).

Distribution and habitat: It is a narrow endemic to China, distributed over the eastern spurs of the Qilian Mountains, in the Tibetan Plateau, in eastern Qinghai Province (e.g., Haixi Mongol and Tibetan Autonomous Prefecture: Ulan County; Hainan Tibetan Autonomous Prefecture: Gonghe and Xinghai counties; Huangnan Tibetan Autonomous Prefecture: Jainca County; Haidong prefecture-level city: Huzhu and Xunhua counties; Xining prefecture-level city: Chengdong District) and central Gansu Province (e.g., Lanzhou prefecture-level city: Anning and Liangzhou districts, Yuzhong County; Baiyin prefecture-level city: Baiyin District and Huining County; Jinchang prefecture-level city: Yongchang County; Zhangye prefecture-level city: Sunan Yugur Autonomous County).

The species is found in stony, muddy habitats; on fine, compact soils or on red soil; in semi-desert steppes; on eroded hillsides; on open or grassy north slopes of dry and hot valleys without shade; among rocks; on steep drop-offs; on gorge edges, braes, and bluffs; and also under scrubs at elevations in the range of 1670–3500 m (see File S1). The flowering season is from late May to June; the fruiting season is from May to July.

## 4. Materials and Methods

### 4.1. Morphological Data

In order to clarify the differences between the species studied, I selected 43 morphological characters, which are listed in Table 1 (see Table S1). The scores of the characters for *I. pandurata* were obtained from numerous detailed images taken by Shunbang Zhao in Chengdong District, Qinghai Province, and images from other localities in the Gansu and Qinghai provinces (sub *I. tigridia*) [19]; from the descriptions of the relevant species available in references [16,28,29,40]; based on direct examinations of the herbarium specimens from K, LE, and NENU; and from the images of well-developed plants in flowering and fruiting stages available in databases [56,57] that represent collective data from the Chinese herbaria and other virtual herbaria (BR, https://www.botanicalcollections.be/#/en/search/specimen, accessed on 13 October 2024; P, https://science.mnhn.fr/institution/mnhn/collection/p/item/search/form?lang=en_US, accessed on 13 October 2024). The herbarium specimens were identified on the basis of the author’s own experience in dealing with *Iris* species. A complete list of these specimens is provided in File S1.

The examination of *I. tigridia* was carried out using the living specimens collected from wild localities (see also [4]); the author’s own observations of herbarium specimens at ALTB, LE, MHA, MW, NS, NSK, TK, UUH, and VBGI; and the images available in Chinese databases [56,57] (see File S1). Since there are no available images of *I. trippneriana* in botanical databases, the taxonomy of the name was based on a comprehensive study of the protologue [46] and the original material deposited at M (Figure 5).

To describe the seed morphology of *I. tigridia*, I used three herbarium specimens collected in Russia: (1) Republic of Tuva, vicinity of Bayan-Kol, 7 July 1975, *M. Lomonosova & T. Grushevskaya 664* [originally in Russian] (NS0011840!, 13 seeds; see http://herb.csbg.nsc.ru:8081/#fuzzy-label, accessed on 13 October 2024); (2) Republic of Buryatia, Selenginsky District, confluence of the Selenga and Chikoy rivers, 17 July 1962, *L.P. Sergiyevskaya s.n*. (TK!, 12 seeds); and (3) Republic of Buryatia, Kudara River basin, 2 July 1934, *P.I. Kursky s.n*. (LE!). For *I. pandurata*, I used the images taken by Shunbang Zhao in Qinghai Province, Xining City, Nanyou Mountain, south of Wenbei Peak (18 seeds; see https://ppbc.iplant.cn/tu/11007337, accessed on 13 October 2024) and two herbarium specimens collected from the city of Lanzhou, Gansu province, China: (1) 67 km north of town, wormwood-cereal-salsola semi-desert, 29 June 1957, *M.P. Petrov s.n*. [originally in Russian] (LE01263727!, 11 seeds; see http://re.herbariumle.ru/01263727, accessed on 13 October 2024); (2) Mount Yuquan, 1670 m, 31 May 1958, *Zhong Buqiu 8060* (PE01013053, 15 seeds; see https://www.cvh.ac.cn/spms/detail.php?id=ef6a803e, accessed on 13 October 2024).

The terminology used in the morphological study is in accordance with that in reference [58]. Measurements were taken using both actual specimens, and those in the images with scale bars. The rhizome diameter, adventitious root diameter, seed length, and seed diameter were measured in the dry state with a digital Vernier caliper Series 532 (Mitutoyo, Aurora, IL, USA).

The morphological features of the seeds were examined by scanning electron microscopy (SEM). Seeds were mounted on aluminum stubs and sputter-coated with gold in a vacuum chamber, Q150T ES (Quorum Technologies Ltd., Lewes, UK). SEM micrographs were taken through a high-resolution field emission scanning electron microscope, MerlinTM (Carl Zeiss, Oberkochen, Germany), at the Joint-Use Center “Biotechnology and Genetic Engineering”, Federal Scientific Center of the East Asia Terrestrial Biodiversity, Far Eastern Branch, Russian Academy of Sciences. The accelerating voltage was set at 5 kV; the emission current was set at 80 pA.

### 4.2. Taxonomy and Distribution

For the nomenclature, the ICN [49] was consulted. Relevant studies in the literature were also analyzed in addition to the protologues and original material for the names under study. Herbarium codes are based on reference [59]. The information on the distribution of *I. pandurata* in the Gansu and Qinghai provinces and on *I. tigridia* was obtained from the herbarium specimens (see File S1) and the databases [19,56,57], including the only record from reference [12] (see https://www.inaturalist.org/observations/208253253, accessed on 13 October 2024) where plants under these names are considered.

## 5. Conclusions

The major problem with understanding the actual species diversity of the genus *Iris* in China, from where many endemic species were described, e.g., *I. anguifuga* Y.T. Zhao & X.J. Xue, *I. bulleyana* Dykes, *I. leptophylla* Lingelsh., and *I. wilsonii* C.H.Wright., is its still unresolved taxonomy. Recently [41], *I. tibetica*, a new endemic to the Gansu and Qinghai provinces, was found. *Iris thoroldii* Baker was reinstated from the synonymy of *I. potaninii* as a distinct species, which is also an endemic to China distributed on the Tibetan Plateau and in adjacent areas [3]. This study has extended the list of species endemic to China by adding *I. pandurata*, which has a narrow distribution range in the Qilian Mountains.

Previously, *I. pandurata* was recognized by only a few researchers who sometimes confused its distribution with that of *I. tigridia*, or erroneously treated *I. pandurata* as a synonym of *I. tigridia*, or cited it under this name. Nevertheless, as the results of my morphological examination show, *I. tigridia* and *I. pandurata* represent two distinct species. *Iris pandurata* can be distinguished from *I. tigridia* on the basis of several morphological characters: the shape of the rhizome, adventitious roots, and aril; the flowering stem structure; and the falls ornamentation. Also, a detailed analysis of herbarium specimens and images of living plants clearly indicates that *I. pandurata* is significantly different from *I. tigridia* in geographical distribution and that *I. pandurata* should be considered an independent species. This study provides an updated nomenclature of *I. pandurata*, including one new synonym, *I. trippneriana*, a previously unplaced and forgotten name.

## Figures and Tables

**Table 1:** Comparison of morphological characters between the *Iris* species examined.

No.	Characters	*I. pandurata*	*I. tigridia*	*I. trippneriana*
1	Leaf remnants’ shape	Straight	Straight	Straight
2	Leaf remnants’ height	2.5–7	2–5	2.7–5.5
3	Rhizome shape	Compact, vertical	Shortly creeping, horizontal	Compact, vertical
4	Adventitious root shape	Evenly thickened, smooth	Unevenly thickened, wrinkled	Evenly thickened, smooth
5	Adventitious root color	Yellowish brown	Yellowish brown	Yellowish brown
6	Adventitious root diameter	0.2–0.4	0.1–0.4	0.2–0.3
7	Rosette leaf shape	Ensiform or subfalcate	Ensiform or subfalcate	Ensiform or subfalcate
8	Rosette leaf apex shape	Narrowly acute, straight	Narrowly acute, straight	Narrowly acute, straight
9	Rosette leaf texture	Tough	Tough	Tough
10	Rosette leaf surface	Glaucous, finely ribbed	Glaucous, finely ribbed	Finely ribbed
11	Rosette leaf length	5–36	10–30	11–32
12	Rosette leaf width	0.1–0.4	0.1–0.6	0.1–0.4
13	Stem branching	Simple	Simple	Simple
14	Stem height	2–31	3–20	7–16
15	Number of cauline leaves	1	1	1
16	Cauline leaf length	6–7	3–10	6.5
17	Number of bracts	2	2	2
18	Number of bracteoles	0–1	0	0–1
19	Bract shape	Lanceolate	Lanceolate	Lanceolate
20	Bract apex shape	Narrowly acute	Narrowly acute	Narrowly acute
21	Bract texture	Membranous	Membranous	Membranous
22	Outer bract length	2.5–6.5	3–5	4.7–6.4
23	Bracteole length	4.5–6.6	Not applicable	4.4–6.6
24	Pedicel length	0.1–0.3	0.1–0.7	[Subsessile]
25	Number of flowers	2	1	2
26	Flower diameter	3.5–6	2.5–6	4–4.5
27	Perianth tube length	1.5–3.5	1.5–2.5	2.5
28	Flower color	Purple to light blue	Blue to violet (white)	Dark violet
29	Fall ornamentation	Unspotted	Spotted	Unspotted
30	Blade of fall shape	Obovate	Obovate	Obovate
31	Fall apex shape	Acute or rounded	Obtuse or rounded, emarginate	Acute
32	Standard shape	Oblong	Oblong	Oblong
33	Standards apex shape	Emarginate or rounded	Emarginate or wavy	Emarginate or rounded
34	Fruit shape	Fusiform	Fusiform	[Ovary fusiform]
35	Fruit length	2.4–3.7	2–4.5	–
36	Fruit width	1–2	1–2	–
37	Seed shape	Pyriform	Pyriform	–
38	Seed appendage	Aril	Aril	–
39	Appendage shape	Beak-shaped	Mushroom-shaped	–
40	Seed color	Reddish brown	Reddish brown	–
41	Seed surface	Glossy, rugose	Glossy, rugose	–
42	Seed length	3.1–4.6 mm	3.2–4.4 mm	–
43	Seed diameter	2–2.8 mm	2.3–3 mm	–

All measurements are in centimeters except for seeds. Data are presented as range (minimum and maximum) of values. En dashes (–) indicate lack of information. See Supplementary raw data in Table S1 for more details.

## Data Availability

All data supporting the reported results are presented as Supplementary Materials.

## Supplementary Materials

The following supporting information can be downloaded at https://www.mdpi.com/article/10.3390/plants13233418/s1, File S1: Herbarium specimens of *Iris pandurata* and *I. tigridia* examined; Table S1: Raw data of the morphological analysis of the *Iris* species studied (the numbers of the morphological characters correspond to those in Table 1).
