# Supplementary material for: Morphological Evidence Supports the Taxonomic Reinstatement of the Endemic Chinese Species Iris pandurata (Iridaceae) by Segregation from I. tigridia
Source: Plants (Basel). 2024 Dec 5;13(23):3418. doi: 10.3390/plants13233418 (PMC11644363; doi:10.3390/plants13233418)
Supplement: Supplementary file 1 [file plants-13-03418-s001.zip › File S1.pdf]

File S1. Herbarium specimens of *Iris pandurata* and *I. tigridia* examined.

(i) **Specimens of *I. pandurata* examined personally at LE** (https://en.herbariumle.ru/?t=occ, accessed on 13 October 2024).

CHINA: China occidentalis, Terra Tangutorum (prov. Kansu), Jugum S. a fl. Tetung, region inferior, ad rupes praeruptas rarissima, [fl.], 17/29 May 1873, *N.M. Przewalski 58* (LE01011518); China occidentalis, Regio Tangut (prov. Kansu), ad affl. fl. Bagagorgi, 9500 f. [2900 m], in abruptis faucium limosorum frequens, [fl.], 26 April / 8 May 1880, *N.M. Przewalski 35* (LE01011519); China occidentalis, Regio Tangut (prov. Kansu), ad fl. Hoangho circa ostium fl. Tschurmyn, 8500 f. [2600 m], fl. violacei v. rubroviolacei, 6/18 May 1880, *N.M. Przewalski s.n.* (LE01011520); China borealis, prov. Kansu occidentali, valle fl. Dshanba, [fl.], 15 April 1885, *G.N. Popanin s.n.* (LE01263733); China borealis, prov. Kansu occidentali, valle fl. Nimbi-muren, in montibus ripae dextrae, in limoso lapodosis [fl.], 18 April 1885, *G.N. Popanin s.n.* (LE01263734); China borealis, prov. Kansu occidentali, Laoquasia [Lovachen], valle fl. Nimbi Muren ad intaoitum angustiarum, in locaibus australibus, solo lapidoso, [fl.], 19 April 1885, *G.N. Popanin s.n.* (LE01263729); China borealis, prov. Kansu occidentali, valle fl. Karyn infra vicum Kashir, [fl.], 5 May 1885, *G.N. Popanin s.n.* (LE01263731); China borealis, prov. Kansu occidentali, valle fl. Liangzhu, [fl.], 8 May 1885, *G.N. Popanin s.n.* (LE01263730); China borealis, prov. Kansu occidentali, ad fl. Nurun-Dshanba, [fl.], 11 May 1885, *G.N. Popanin s.n.* (LE01263728, LE01263732); Gansu, Lanzhou, 67 km north of town, wormwood-cereal-salsola semi-desert, 29 June 1957, *M.P. Petrov s.n.* [originally in Russian] (LE01263727).

(ii) **Digital specimens of *I. pandurata* those available in the Chinese Virtual Herbarium** (https://www.cvh.ac.cn/index.php, accessed on 4 May 2024) **and National Specimen Information Infrastructure** (indicated by asterisk; see http://www.nsii.org.cn, accessed on 4 May 2024) **databases**, and other herbaria (BR, E, K, NENU, and P) **are labelled *I. pandurata* and *I. tigridia***. Herbarium codes are given in brackets; aor the specimens deposited at HNWP inventory numbers are indicated.

CHINA: Gansu Province: *T.P. Wang 12160* (PE01013055); China occidentalis, regio Tangut (prov. Kansu), 1880, *N.M. Przhevalsky s.n.* (K000499075!, det. W.R. Dykes); China occidentalis, Regio Tangut (prov. Kansu), ad fl. Hoangho circa ostium fl. Tschurmyn, 8500 f., fl. violacei v. rubroviolacei, 6/18 May 1880, *N.M. Przewalski s.n.* (PE00034030); China borealis, prov. Kansu occidentali, 1885, *G.N. Potanin s.n.* (BR0000033071534, E, P02162658); Lanzhou City, Yuquan Mountain, 1670 m, 31 May 1958, *8060* (HNWP No. 002937, PE01013053, PE01013054); Sunan County, Jiutiaoling in Qilian Mountains, 2300 m, 30 June 1959, *3607* (NENU No. 159659!); Baiyin City, Dijiatai, 15 August 1959, *6224* (NENU No. 158125!); Huining County, 2000 m, 19 May 1964, *64720* (LZU00874995*); Baiyin City, 2000 m, 26 April 1965, *64722* (LZU00874986*); Lanzhou City, Renshou Mountain, 25 April 1985, *85005* (LZD0003114). **Qinghai Province**: Very occasional on the hottest loess bluffs above Tien Tang Suu, 9000, [fl.], 25 May [1915], [*R. Farrer & W. Purdom*] *498* (E!); Xining, hillside, 2300 m, 26 June 1958, *8175* (HNWP No. 0218533, PE01013058, PE01013059); 12 June 1959, *4051* (LZD0003115, LZD0003116); Gonghe County, 17 June 1960, *0292* (PE01013056, PE01013057); Xinghai County, Heka Township, 3300 m, 28 June 1965, *00110* (NAS00555433*, NENU No. 290207!, HNWP No. 12042, NWTC0093976*); Xinghai County, Heka Township, Yangqutai, 2650 m, 21 May 1966, *019* (NENU No. 290454!); Xining City, 3000 m, 16 June 1970, *712* (HNWP No. 22014); Huangnan Prefecture, Jainca County, 25 June 1972, *10000* (HNWP No. 32110); Ulan County, Tongpu Town, Haerhate Village, 3200–3500 m, 10 July 1981, *0112* (HNWP No. 100011); Xining, Dayou mountain, 2412 m, 17 May 1989, *1028* (HNWP No. 156635); Xining, 2446 m, 17 May 1989, *0504* (HNWP No. 156535); Xining, Dayou Mountain, 2446 m, 17

May 1989, *0505* (HNWP No. 156536); Xining, 2446 m, 17 May 1989, *0506* (HNWP No. 156537); Xining, Nan mountain, 2340 m, 05 June 1989, *1044* (HNWP No. 156645); 2277 m, 08 May 1990, *5662* (HNWP No. 160437); Xiang Hua County, Daewei [Daoweixiang], open eroded hillside, red soil, 2370 m, 14 June 2000, *SQAE 44* (E00287868!, sub "*Iris songarica* Schrenk"); Xining, 36°37'14"N 101°45'22"E, 2500 m, 24 August 2008, *zy0018* (PE02238508).

(iii) **Digital specimens of *I. tigridia* those available in the Chinese Virtual Herbarium and National Specimen Information Infrastructure databases.** The list preferably includes information on elevation, collecting date and number, and unique specimen identifier (for the specimens deposited at HNWP inventory numbers are indicated).

CHINA: 14 April 1918, *3662* (TIE00042601); 23 May 1931, *T.N. Liou 2064* (PE01013755). **Beijing:** 28 April 1951, *15048* (PE01013747, PE01013748); 04 May 1951, *15074* (PE01013758). **Hebei Province:** *s.n.* (PE01013739); *s.n.* (PE01013740); 23 May 1927, *E. Licent 7241* (TIE00042602); Hsiaowutaishan, Chark'ar, 26 May 1930, *H.W. Kung 55* (PE01013769); Hsiaowutaishan, 1932, *2620* (PEY0051856); July 1936, *37503* (PE01013743); 12 July 1936, *Wu & Yang 37129* (PE01013760); 1951, *2013* (PE01013751, PE01013754); 1951, *2014* (PE01013752, PE01013753); 1951, *2023* (PE01013750); 1951, *2101* (PE01013762); 2 April 1951, *85* (PE01013759); 12 May 1951, *15165* (PE01013745); 12 May 1951, *15171* (PE01013746, PE01013749); [Xinglong County] vicinity of Hsinglung Hsien City, 20 May 1951, *Y. Liu 15363* (PE01013761); 9 May 1952, *72* (PE01013744); 8 May 1956, *20* (PEY0051852–PEY0051855); 1500 m, 17 August 1959, *8417* (PE01013741, PE01013742); Zhuolu County, 1278 m, 12 May 2014, *SZ3497* (PE02031955, PE02031979); Zhuolu County, 1278 m, 12 May 2014, *SZ3498* (PE02015953, PE02015959, PE02034842). **Inner Mongolia Autonomous Region:** 3 May 1924, *S.J. Licent 7241* (PE01013756); Horqin Right Front Banner, 10 June 1950, *168* (IFP15405021x0039, IFP15405021x0040); Manzhouli City, 800 m, 26 June 1951, *954* (IFP15405021x0028, IFP15405021x0029, IFP15405021x0030, IFP15405021x0031); 6 July 1952, *226* (PE01013738); Yakeshi City, 700 m, 2 July 1954, *2103* (IFP15405021x0041); 1955, *s.n.* (PE01013737); Hulunbuir, 11 May 1958, *185* (IBSC0629088); Hailar City, 5 July 1958, *20* (IFP15405021x0032); Hailar City, 11 July 1958, *20* (IFP15405005a0082, IFP15405005a0083, IFP15405021x0033, IFP 15405021x0034, IFP15405021x0035); New Barag Left Banner, 15 May 1959, *3* (IFP15405021x0036, IFP15405021x0037, IFP15405021x0038); 14 June 1959, *17* (NAS00555564); Aershan City, 8 July 1963, *2522* (IFP15405021x0043); Baotou City, 2100 m, 16 July 1964, *50* (PE01013757); Keshiketeng, 25 July 1973, *367* (IFP15405021x0024); Hulunbuir City, 16 May 1986, *86-0023* (JCE00079971); Horqin Right Front Banner, 27 May 1987, *362* (IFP15405021x0042); Zhenglan Banner, 42°41'26.5"N 116°31'54.5"E, 1375 m, 21 May 2007, *654* (PE01953305); Chifeng City, 4 May 2008, *s.n.* (BNU0012978); Hulunbuir City, 1 September 2013, *X.W. Huang 1939* (KUN1447845); Hohhot City, Lin County, 7 May 2015, *040* (JCE00077380); Tuyou, Baotou City, 1525 m, 7 May 2015, *165* (JCE00076417, JCE00077382); Liangcheng County, Ulanqab City, 9 May 2015, *196* (JCE00076415); Liangcheng County, Ulanqab City, 1778 m, 9 May 2015, *222* (JCE00076419); Tuyou, Baotou City, 16 May 2015, *155* (JCE00077381); Daqingshan, Hohhot City, 1627 m, 17 May 2015, *274* (JCE00077448); Wuchuan County, Hohhot City, 1781 m, 17 May 2015, *319* (JCE00076421); Zhuozi County, Ulanqab, 22 May 2015, *108* (JCE00076422); Zhuozi County, Ulanqab City, 1390 m, 22 May 2015, *145* (JCE00076413, JCE00077451); Xinghe County, Ulanqab City, 1809 m, 23 May 2015, *183* (JCE00077379); Xinghe County, Ulanqab City, 23 May 2015, *214* (JCE00076418); Xinghe County, Ulanqab City, 1995 m, 23 May 2015, *215* (JCE00076414); Hulunbuir City, 525 m, 18 May 2016, *0340* (IFP0234331, IFP0235414); Arong, 309 m, 20 May 2016, *048* (YAK0007084); Hulunbuir City, 596 m, 30 May 2016, *0378* (IFP0234365, IFP0235446, IFP0236371); Guyang County, 2135.8 m, 9 May 2018, *150222180509013LY* (00010762); Hulunbuir, Ergun City, 519 m, 14 May 2018, *1179* (IFP0254574, IFP0255824, IFP0256982); Hulunbuir, Ergun City, 550 m, 14 May 2018, *1188* (IFP0254583, IFP0255831,

IFP0256989); Hulunbuir City, 631 m, 18 May 2018, *1255* (IFP0254650, IFP0255896, IFP0257054); Ulanqab, 41°10'36.7"N 112°34'53.7"E, 2060 m, 7 June 2018, *150927180607052LY* (IATM0000555); 1644 m, 9 June 2018, *150123180609011LY* (IATM0000064); Aershan, 920 m, 10 June 2018, *157* (YAK0007666); Xilinhot City, 1183 m, 17 May 2019, *152502190517030LY* (IATM0004688). **Jilin Province:** Shuangliao City, 30 May 1932 (IFP15405021x0026); Panshi County, Jilin City, 43°17'44.89"N 125°53'34.63"E, 360 m, 20 June 2016, *AnHC038* (KUN1441922). **Liaoning Province:** Shenyang City, 4 June 1957, *596* (IFP15405021x0001, IFP15405021x0002, IFR15405021x0003); Lingyuan City, 24 April 1960, *184* (IFP15405021x0020, IFP15405021x0021); Jianping County, 27 April 1960, *207* (IFP15405011x0037, IFP15405021x0004, IFP15405021x0005, IFP15405021x0006); Jianchang County, 2 May 1961, *96* (IFP15405021x0046); Jianchang County, 2 May 1961, *104* (IFP15405021x0045); Dalian City, 27 April 1966, *1090* (IFP15405021x0011, IFP15405021x0012); Dalian City, 27 April 1966, *1094* (IFP15405021x0010); Fuxin, 3 May 1966, *73* (IFP15405021x0013); Tieling County, 9 May 1974, *2914* (IFP154050210a001, IFP15405021x0016, IFP15405021x0017, IFP15405021x0018, IFP15405021x0019); Tieling County, 9 May 1974, *2916* (IFP15405021x0014, IFP15405021x0015); Beipiao City, 7 May 1984, *66* (NEFI115001003002001); Yixian, 14 May 1984, *749* (IFP15405021x0007, IFP15405021x0008, IFP15405021x0009); Tieling City, May 1985, *B230* (SYAUF007097); Tieling City, May 1985, *B231* (SYAUF007098); Tieling, 5 May 1985, *291* (MAU001927); Beining City, 20 April 1989, *1780* (IFP15405021x0022, IFP15405021x0023, IFP15405021x0024); Jianping County, Chaoyang City, 797 m, 24 July 2015, *676* (IFP0258897); Anshan City, 136 m, 14 May 2016, *AS-P001-092* (SYAUF001671); Anshan City, 136 m, 14 May 2016, *AS-P001-093* (SYAUF001676); 10 May 1918, *M. Miura* (NAS00555563). **Shanxi Province:** *5059* (HNWP No. 8204); 1929, *T. Tang 918* (PE01013770); 2500 m, 14 June 1956, *524* (PE01013768); 2200 m, 28 June 1957, *1464* (PE01013763); Qin County, 11 May 1959, *00110* (HNWP No. 8190, PE01013766, PE01013767); 11 May 1959, *00150* (HNWP No. 8189); Qin County, 11 May 1959, *110* (HSIB020561); 12 May 1959, *00150* (PE01013764, PE01013765); Ningwu County, 8 June 1959, *15046* (HNWP No. 8203, HNWP No. 8206, HSIB020563). **MONGOLIA:** 15 May 1999, *99-025* (PE01679968); 21 May 1999, *99X047* (PE01679969).
